# Triple Vector-Borne Co-infection During Pregnancy in the United Arab Emirates: A Case Report at 21 Weeks’ Gestation

**DOI:** 10.7759/cureus.116305

**Published:** 2026-09-15

**Authors:** Fathimanidha Faslin, Shalini Fernandes, Komal Hazari

**Affiliations:** 1 Department of Obstetrics and Gynecology, Latifa Women and Children Hospital, Dubai, ARE; 2 Department of Obstetrics and Gynecology, Dubai Medical College for Girls, Dubai, ARE; 3 Department of Obstetrics and Gynecology, Mohammed Bin Rashid University of Medicine and Health Sciences, Dubai, ARE; 4 Department of Obstetric Medicine, Dubai Health, Dubai, ARE

**Keywords:** co- infection, dengue fever, malaria, pregnancy, : vector-borne

## Abstract

Pregnancy is a unique physiological state characterised by altered immune responses that may predispose women to severe manifestations of infection. Vector-borne diseases such as malaria and dengue pose significant risks to pregnant women and their foetuses, including miscarriage, preterm labour, placental abruption, and intrauterine growth restriction (IUGR). Co-infection with multiple vector-borne pathogens is extremely rare but can exacerbate maternal morbidity and complicate foetal outcomes. This case report aims to describe the clinical presentation, diagnosis, management, and maternal and foetal outcomes of a rare triple vector-borne co-infection during pregnancy.

A 32-year-old pregnant woman at 21 weeks of gestation presented with a triple vector-borne infection involving *Plasmodium vivax*, *Plasmodium falciparum*, and dengue virus. Prompt diagnosis was achieved through rapid diagnostic testing for dengue and malarial parasites, followed by antimalarial therapy and symptomatic supportive treatment. The patient showed complete clinical and laboratory recovery, evidenced by improvement in haemoglobin and platelet counts, normalization of liver function parameters, and clearance of parasitaemia.

This case highlights the diagnostic and therapeutic challenges associated with multiple vector-borne infections during pregnancy and illustrates the potential value of early recognition, appropriate diagnostic evaluation, multidisciplinary management, and close maternal and foetal monitoring in achieving a favourable clinical outcome.

## Introduction

Vector-borne diseases remain a substantial global health concern, accounting for over 17% of infectious diseases and causing more than 700,000 deaths annually [1]. Malaria and dengue, among the most prevalent vector-borne diseases, are independently associated with significant maternal and foetal complications during pregnancy, including anaemia, miscarriage, preterm labour, intrauterine growth restriction (IUGR), and stillbirth [1]. Pregnancy is a unique physiological state characterised by immunological and physiological adaptations that may increase susceptibility to severe manifestations of infection [2].

During pregnancy, maternal immunity undergoes changes that have traditionally been described as a shift from predominantly T-helper 1 (Th1)-mediated cellular immunity towards a T-helper 2 (Th2)-predominant response [3]. This shift has been particularly described during the second trimester and contributes to maternal tolerance of the foetus; however, it may also increase susceptibility to certain intracellular pathogens and favour parasite persistence [3]. These immunological adaptations may contribute to the increased susceptibility to malaria observed during pregnancy [3].

When malaria and dengue occur concurrently in the same individual, the severity and duration of one or both infections may increase [4]. Malaria has a complex pathophysiology involving direct RBC destruction, as well as inflammatory and immune responses, with anaemia being an important clinical manifestation [4]. Dengue similarly involves complex interactions between the virus and the host immune response and is frequently associated with thrombocytopenia [4]. Consequently, concurrent infection may result in overlapping and potentially more severe clinical manifestations than either infection alone, making early recognition and appropriate management particularly important [4].

Although malaria-dengue co-infection has been reported, concurrent infection with *P. falciparum*, *P. vivax*, and dengue virus during pregnancy remains rare and poorly documented. Physiological changes during pregnancy, including altered immune responses and increased plasma volume, may further complicate the clinical presentation and management of these infections [5].

This report aims to describe the clinical presentation, diagnostic challenges, management, and short-term maternal and foetal outcomes of a rare triple vector-borne co-infection with *P. falciparum*, *P. vivax*, and dengue virus in a pregnant patient at 21 weeks of gestation. The case also highlights the importance of considering concurrent vector-borne infections in febrile pregnant patients with recent travel to endemic regions.

## Case presentation

This single-patient case report describes a rare triple vector-borne co-infection during pregnancy and is presented for its educational value in illustrating the diagnostic and therapeutic challenges associated with simultaneous multi-pathogen infections in pregnancy.

Clinical presentation

A 32-year-old primigravida at 21 weeks of gestation presented to our hospital in the United Arab Emirates (UAE) with a two-day history of fever, fatigue, and episodes of coffee-ground vomiting, along with one episode of syncope. She had recently returned from Ethiopia, where she had stayed for approximately 25 days, and had arrived back in the UAE five days before presentation. During her stay in Ethiopia, she reported exposure to mosquitoes and had not received antimalarial prophylaxis before or during travel.

On admission, her blood pressure was 90/50 mmHg, pulse rate was 95 beats/min, oxygen saturation was 99% on room air, and body temperature was 36.3°C. On examination, she was icteric. Respiratory and cardiovascular examinations were otherwise unremarkable.

On abdominal examination, the abdomen was soft with generalized tenderness, which was more pronounced in the right upper quadrant. Obstetric examination revealed a gravid uterus corresponding to approximately 20-21 weeks of gestation, and foetal heart sounds were detected.

Diagnostic assessment

Initial laboratory investigations demonstrated severe anaemia, with a haemoglobin level of 6.2 g/dL; thrombocytopenia, with a platelet count of 29,000/µL; hypokalaemia; hypocalcaemia; elevated liver enzymes; and hyperbilirubinaemia. The detailed laboratory findings and subsequent trends are presented in Table 1.

**Table 1:** Trends in laboratory parameters from admission to day 4. ALT: Alanine aminotransferase; AST: Aspartate aminotransferase.

Parameter	Reference range and units	On admission	Day 2	Day 3	Day 4
Haemoglobin	12.0-15.0 g/dL	6.2	6.9	8.1	8.4
Platelets	150-410 × 10³/µL	29	45	64	91
ALT	0-31 U/L	47	24	24	26
AST	0-32 U/L	85	Not tested	52	47
Bilirubin	0-1.0 mg/dL	3.03	3.15	2.41	1.93
CRP	<5.0 mg/L	200.5	181	142	91
Procalcitonin	<0.05 ng/mL	91.24	60.2	13.8	7.58
Calcium	8.9-10.2 mg/dL	6.9	7.2	Not tested	8.2
Potassium	3.3-4.8 mmol/L	3.1	3.1	3.5	4.3

Rapid diagnostic testing for malaria and dengue was positive, with the dengue non-structural protein 1 (NS1) antigen test yielding a positive result. Peripheral blood smear microscopy demonstrated *P. falciparum* rings, *P. vivax* rings, and *P. vivax* gametocytes, confirming mixed malarial infection. Obstetric USG demonstrated a single live intrauterine foetus corresponding to 21 weeks of gestation, as shown in Figure 1.

**Figure 1:**
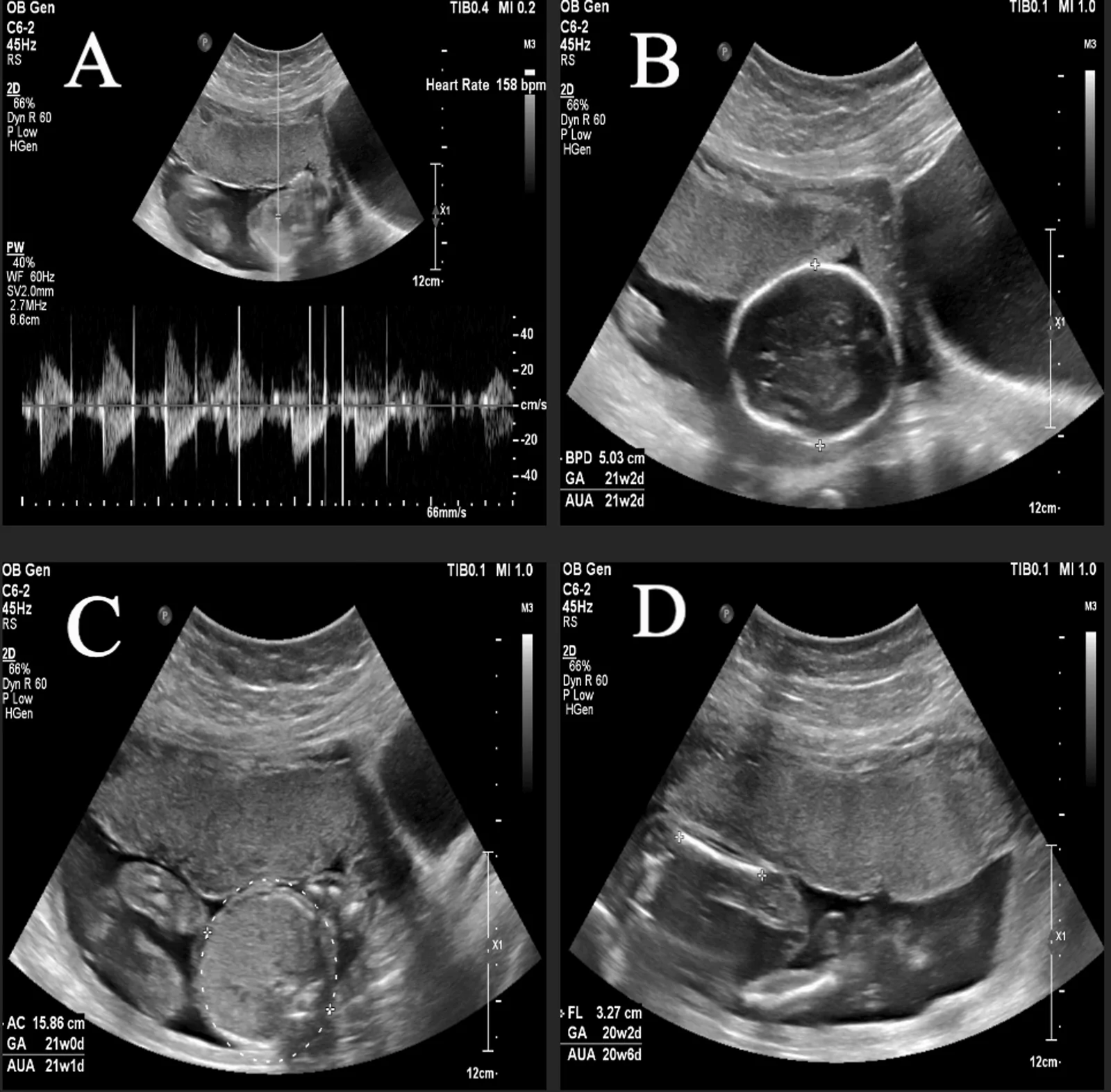
Obstetric ultrasound findings consistent with a single intrauterine fetus at 21 weeks of gestation. (A) Fetal heart rate of 158 bpm. (B) Biparietal diameter corresponding to 21 weeks of gestation. (C) Abdominal circumference corresponding to 21 weeks of gestation. (D) Femur length corresponding to 20 weeks of gestation.

The patient was admitted to the ICU because of severe anaemia, thrombocytopenia, and concurrent infection with *P. falciparum*, *P. vivax*, and dengue virus.

Therapeutic intervention

Management involved a multidisciplinary team comprising internal medicine physicians, infectious disease specialists, obstetricians, cardiologists, and general surgeons. The patient was placed on continuous cardiac monitoring. Supportive treatment included IV fluids, high-flow nasal oxygen, antipyretics, proton pump inhibitor therapy, and foetal monitoring. Fluid input and output were closely monitored, with urine output maintained above 60 mL/hour.

Electrocardiography demonstrated a prolonged corrected QT (QTc) interval of 493 ms (Figure 2). On admission, the serum calcium level was 6.9 mg/dL (reference range: 8.9-10.2 mg/dL), indicating hypocalcaemia, while the serum magnesium level was 1.98 mg/dL (reference range: 1.6-2.6 mg/dL), which was within the normal range. Cardiology consultation was obtained before initiating IV quinine because of the potential risk of ventricular arrhythmias. The patient was maintained on continuous cardiac monitoring, medications associated with QTc prolongation were avoided, and electrolyte levels were closely monitored and corrected as required.

**Figure 2:**
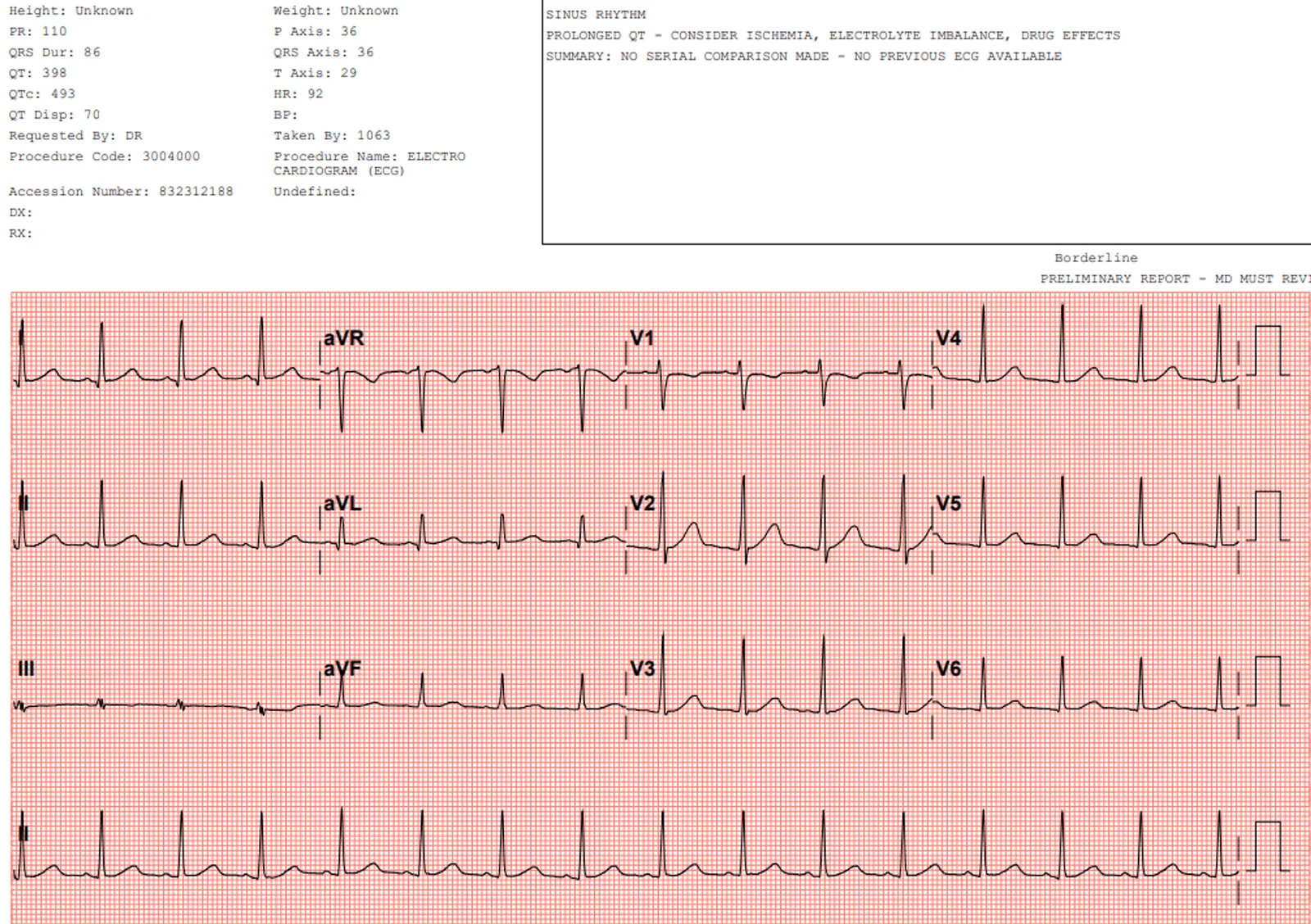
Electrocardiogram showing a prolonged QTc interval.

Specific antimalarial treatment consisted of IV quinine 600 mg infused over four hours every eight hours and IV clindamycin 600 mg every eight hours for seven days. The patient was maintained on continuous cardiac monitoring because of the prolonged QTc interval and the potential for quinine-associated cardiac adverse effects. The clinical course was complicated by quinine-associated hypoglycaemia and tinnitus. Hypoglycaemia was managed with a 10% dextrose infusion. The patient received four units of packed red blood cells and three units of platelets in the context of severe anaemia, thrombocytopenia, and suspected gastrointestinal bleeding, as indicated by coffee-ground vomiting. The general surgery team was consulted to evaluate other potential causes of suspected gastrointestinal bleeding. Tranexamic acid was administered intravenously at a dose of 1 g three times daily.

Complete blood count, coagulation profile, liver function tests, blood glucose, electrolytes, and parasitaemia were monitored during treatment. Electrocardiography was repeated every 24-48 hours to monitor for arrhythmias. Potassium was supplemented intravenously and maintained above 4 mmol/L. Magnesium and calcium levels were monitored and supplemented as required. The patient was also monitored closely for bleeding manifestations.

A dietitian was consulted, and a high-iron diet and nutritional supplementation with Fortisip 125 mL were prescribed.

Follow-up and outcomes

The patient demonstrated progressive clinical and haematological improvement during treatment. Haemoglobin increased from 6.2 g/dL on admission to 8.4 g/dL by day 4, while the platelet count increased from 29,000/µL to 91,000/µL. Bilirubin decreased from 3.03 mg/dL on admission to 1.93 mg/dL by day 4, and inflammatory markers also showed a downward trend.

Parasitaemia decreased during antimalarial treatment, reaching <0.1% by day 4, as illustrated in Figure 3. The patient was transferred from the ICU to the general ward on day 5, completed seven days of antimalarial therapy, and was discharged in stable condition after a total hospital stay of seven days. Follow-up was arranged for continued monitoring of maternal and foetal well-being throughout the remainder of the pregnancy.

**Figure 3:**
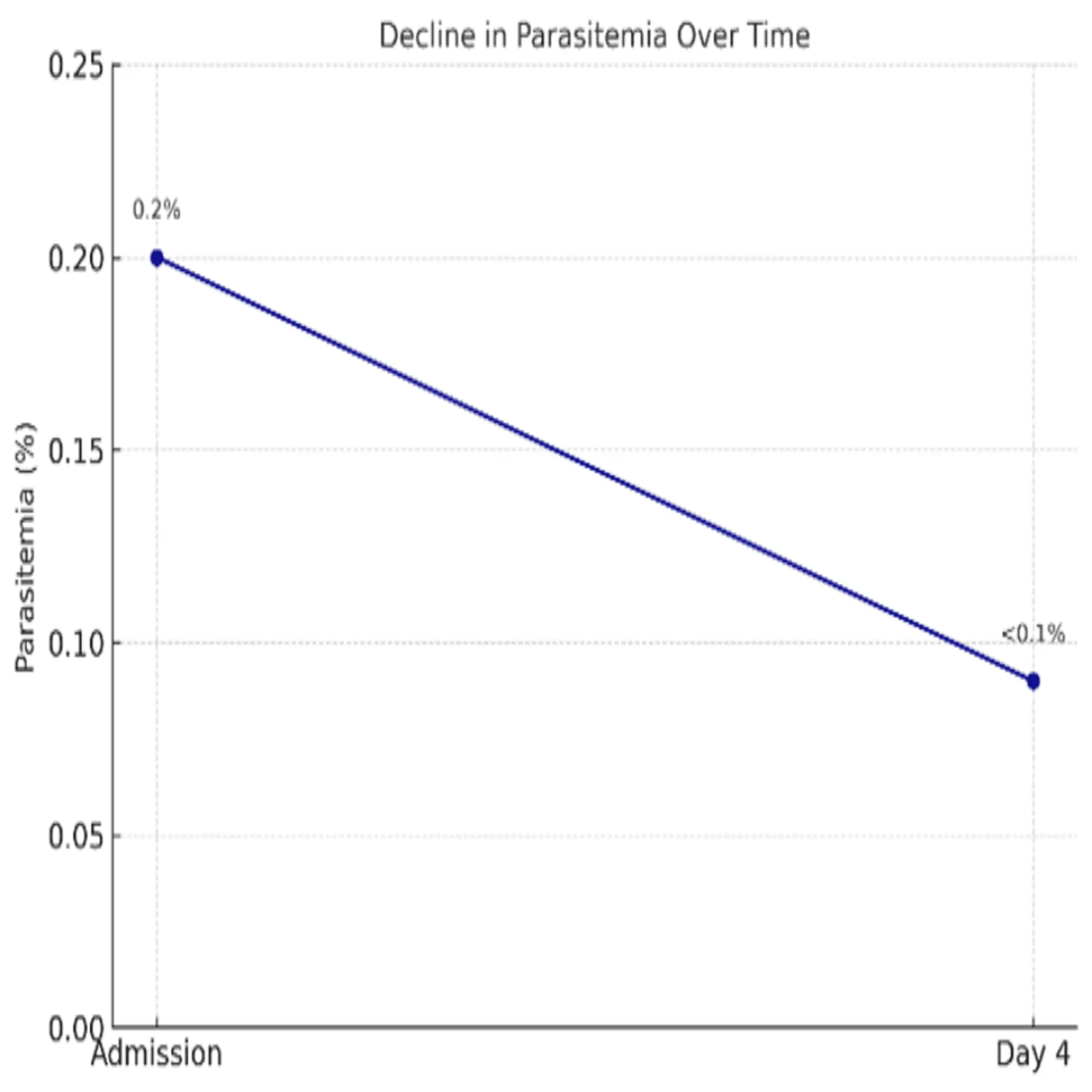
Line graph showing the decline in parasitaemia (%) from admission to day 4 following antimalarial treatment. Day 4 parasitaemia was estimated at <0.1%.

## Discussion

Triple vector-borne co-infection involving *P. falciparum*, *P. vivax*, and dengue virus in pregnancy is exceedingly rare, with only isolated case reports documented in the literature. The present case adds to the limited body of evidence and highlights the importance of early diagnosis and prompt multidisciplinary management to achieve favourable maternal and foetal outcomes. The overlap in the geographic distribution of these pathogens, especially in endemic regions, creates the potential for simultaneous transmission; however, the clinical documentation of such triple infections remains limited [1,6].

Malaria and dengue are independently associated with significant maternal and foetal morbidity. *P. falciparum* is known for its ability to cause placental sequestration, contributing to IUGR, stillbirth, and preterm labour. Although *P. vivax* is considered less virulent, it can still lead to severe anaemia, a major contributor to maternal morbidity and mortality during pregnancy [2]. Dengue virus infection can result in plasma leakage, thrombocytopenia, and haemorrhagic complications, which are particularly hazardous in the obstetric context [7].

In the present case, the coexistence of these three infections may have had a synergistic effect. Co-infection may exacerbate systemic inflammation through heightened cytokine responses, contributing to endothelial dysfunction, maternal haemodynamic instability, and foetal hypoxia [8]. The resulting complications, including severe anaemia, thrombocytopenia, and GI bleeding, require intensive monitoring and supportive therapy, including packed RBC and platelet transfusions when clinically indicated.

The diagnosis of co-infections is particularly challenging because of overlapping clinical features, such as fever, myalgia, and thrombocytopenia. In this case, early identification relied on rapid diagnostic testing, followed by peripheral blood smear microscopy for confirmation of the diagnosis. However, in many settings, delayed or missed diagnoses occur because of the limited availability of additional diagnostic modalities, such as PCR or enzyme-linked immunosorbent assay (ELISA), for the detection of dengue and malaria [5, 9]. The need for improved access to integrated serological and molecular diagnostic tools is critical, especially in resource-limited, high-burden regions [7].

The management of co-infections during pregnancy requires a careful balance between efficacy and foetal safety. Antimalarial therapy with quinine and clindamycin was chosen because of its relative safety in the second trimester, although the patient experienced quinine-induced hypoglycaemia and tinnitus. Dengue management is supportive, focusing on careful fluid management and transfusion when clinically indicated to address bleeding and haemorrhagic risk. Close multidisciplinary care, including foetal surveillance, contributed to favourable short-term maternal and foetal outcomes.

This case highlights important public health implications. Historically, dengue cases in the UAE have been largely imported. However, since 2023, locally transmitted cases with no travel history have been reported, likely driven by climate change and increasingly favourable conditions for vector proliferation. This evolving epidemiological landscape emphasises the need for enhanced vector surveillance, preventive strategies, and heightened clinical suspicion in pregnant patients with febrile illnesses who have recently travelled to endemic regions [1,6,10]. Previously reported cases of similar triple vector-borne co-infections during pregnancy are summarized in Table 2 [11].

**Table 2:** Summary of reported cases of triple vector-borne infections during pregnancy.

Author/Year	Country	Gestational Age	Pathogens	Clinical Features	Treatment	Maternal Outcome	Foetal Outcome
Pande A and Guharoy D (2013) [11]	India	6 months	*Plasmodium falciparum*, *Plasmodium vivax*, dengue virus	Fever, severe anaemia, thrombocytopenia, and rash	Artesunate-based combination therapy, supportive care, and packed RBC transfusion	Recovered	Discharged with a live foetus; ongoing follow-up
Current case	UAE	21 weeks	*Plasmodium falciparum*, *Plasmodium vivax*, dengue virus	Fever, severe anaemia (Hb 6.2 g/dL), thrombocytopenia, and coffee-ground emesis	IV quinine and clindamycin, packed RBC and platelet transfusions, and ICU care	Recovered	Live intrauterine foetus at discharge; longer-term outcome unavailable

Overall, this case emphasises the complexity of diagnosing and managing multiple vector-borne infections during pregnancy and supports the importance of integrated diagnostic protocols, multidisciplinary care, and preventive public health interventions to mitigate the risks in vulnerable populations.

Limitations

First, this report describes a single patient and therefore cannot establish causal relationships between the concurrent infections or determine whether their clinical manifestations were synergistic or additive. Second, the period of direct clinical observation was limited to the seven-day hospitalization. Although follow-up was arranged after discharge, the patient did not return to our institution; therefore, longer-term maternal and foetal outcomes were unavailable.

## Conclusions

The coexistence of *P. vivax*, *P. falciparum*, and dengue virus has rarely been reported in the literature, with only a few cases describing similar triple vector-borne co-infections. This case highlights the complexity of managing triple vector-borne infections during pregnancy, emphasising the importance of awareness, early diagnosis, and specialised care. A detailed travel and exposure history, together with timely malaria testing, peripheral blood smear examination, and dengue-specific testing, can facilitate the recognition of co-infection when clinical manifestations overlap. A tailored multidisciplinary approach is essential to minimise maternal and foetal risks. Increased awareness of co-infections in patients travelling from endemic areas is crucial for improving outcomes. Appropriate diagnostic and treatment protocols should be followed. This case also highlights the need for public health interventions to minimise vector exposure during outbreaks. The limited reports of triple vector-borne infections during pregnancy emphasise the rarity of this case.
